# Development and Feasibility Testing of an mHealth (Text Message and WeChat) Intervention to Improve the Medication Adherence and Quality of Life of People Living with HIV in China: Pilot Randomized Controlled Trial

**DOI:** 10.2196/10274

**Published:** 2018-09-04

**Authors:** Yan Guo, Zhimeng Xu, Jiaying Qiao, Y Alicia Hong, Hanxi Zhang, Chengbo Zeng, Weiping Cai, Linghua Li, Cong Liu

**Affiliations:** ^1^ Department of Biostatistics and Epidemiology School of Public Health Sun Yat-sen University Guangzhou China; ^2^ Sun Yat-sen Center for Migrant Health Policy Sun Yat-sen University Guangzhou China; ^3^ Sun Yat-sen Global Health Institute Institute of State Governance Sun Yat-sen University Guangzhou China; ^4^ Department of Health Promotion and Community Health Sciences School of Public Health Texas A&M University College Station, TX United States; ^5^ Department of Infectious Disease Eighth People's Hospital Guangzhou China

**Keywords:** mHealth, social media, medication adherence, people living with HIV, randomized controlled trial

## Abstract

**Background:**

Most people living with HIV (PLWH) reside in middle- and low-income countries with limited access to health services. Thus, cost-effective interventions that can reach a large number of PLWH are urgently needed.

**Objective:**

The objective of our study was to assess the feasibility and acceptability of an mHealth intervention among PLWH in China.

**Methods:**

Based on previous formative research, we designed an mHealth intervention program that included sending weekly reminders to participants via text messages (short message service, SMS) and articles on HIV self-management three times a week via a popular social media app WeChat. A total of 62 PLWH recruited from an HIV outpatient clinic were randomly assigned to intervention or control group. The intervention lasted for 3 months, and all participants were assessed for their medication adherence, presence of depression, quality of life (QoL), and CD4 (cluster of differentiation 4) counts. Upon completing the intervention, we interviewed 31 participants to further assess the feasibility and acceptability of the study.

**Results:**

At baseline, the intervention and control groups did not differ in terms of demographic characteristics or any of the major outcome measures. About 85% (53/62) of the participants completed the intervention, and they provided valuable feedback on the design and content of the intervention. Participants preferred WeChat as the platform for receiving information and interactive communication for ease of access. Furthermore, they made specific recommendations about building trust, interactive features, and personalized feedback. In the follow-up assessment, the intervention and control groups did not differ in terms of major outcome measures.

**Conclusions:**

This pilot study represents one of the first efforts to develop a text messaging (SMS)- and WeChat-based intervention that focused on improving the medication adherence and QoL of PLWH in China. Our data indicates that an mHealth intervention is feasible and acceptable to this population. The data collected through this pilot study will inform the future designs and implementations of mHealth interventions in this vulnerable population. We recommend more innovative mHealth interventions with rigorous designs for the PLWH in middle- and low-income countries.

**Trial Registration:**

Chinese Clinical Trial Registry ChiCTR1800017987; http://www.chictr.org.cn/showprojen.aspx?proj=30448 (Archived by WebCite at http://www.webcitation.org/71zC7Pdzs)

**Registered Report Ientifier:**

RR1-10.2196/

## Introduction

Most people living with HIV (PLWH) reside in middle- and low-income countries [1]. Delivering effective interventions to this vulnerable and stigmatized population remains a critical public health challenge. In recent years, mobile-based interventions, or mHealth interventions, have emerged as a promising solution to deliver health services to PLWH. For example, the WelTel program in Kenya sent short message service (SMS) text messages to PLWH, and this resulted in improved antiretroviral therapy (ART) adherence [2]. A recent Cochrane review has identified 17 SMS text message interventions to promote medication adherence, most of which have shown initial potential [3]. However, of the existing mHealth interventions that promote ART adherence among PLWH in middle- or low-income countries, only few have employed a rigorous design (eg, randomized controlled trial, RCT) or assessed the quality of life (QoL) measures and clinical outcomes such as CD4 (cluster of differentiation 4) counts of these people.

More than 64% of the world’s population owns a mobile phone, and in several high- and middle-income countries, a majority of the population owns a smartphone [4]. Because of this, there has been a growing interest in delivering mHealth interventions via social media such as Facebook and mobile apps [5-8]. In a recent review of social media interventions that deliver HIV services, out of the 26 studies identified, 18 were conducted in high-income countries, 8 in middle-income countries, and none in low-income countries. Furthermore, of the 26 studies, only 1 was designed to improve ART adherence and none to promote the QoL of PLWH [8].

As the most populous country in the world, China has 1.3 billion mobile phone users, with a 95% penetration rate [9]. More than 740,000 PLWH live in China; they face a high level of stigma, and the prevalence of depression in this group is high [10]. Although Facebook and Twitter are not accessible in China, Chinese people are active on other social media platforms. With more than 570 million users, WeChat is the most popular social media platform in China; 93% of the residents in the major cities of China log into WeChat every day [11]. Recently, WeChat-based behavioral interventions have shown feasibility and acceptability [12,13]. The high ownership rates of mobile phones and wide popularity of WeChat suggest a promising platform to deliver low-cost interventions to the stigmatized population of PLWH. Although HIV-related mHealth interventions have amassed growing interests and have shown initial potential globally [5,6,8], the use of such programs has been limited in China despite the high rates of mobile phone use and access to social media. Recent reports on mHealth interventions for HIV-affected populations in China were either study protocols [14,15] or the delivery of SMS text messages only [16]. There are few mHealth interventions for PLWH in China that have been tested with rigorous design. Accordingly, we developed one of the first mHealth (WeChat+SMS text message) interventions to improve the ART adherence and QoL of PLWH in China and pilot-tested its feasibility and acceptability via an RCT. We hypothesized that the mHealth intervention would be feasible for and acceptable to PLWH in China.

## Methods

### Study Setting

This study was conducted in a hospital that has been offering services to PLWH in a large metropolitan area in South China from October 2016 to March 2017. The hospital serves more than 14,000 patients with HIV in the region.

### Intervention Program

The development of the intervention program was guided by the information-motivation-behavioral skills model [17], the literature of mHealth interventions, and our formative research. The initial intervention protocol was developed based on prior mHealth interventions to improve medication adherence in PLWH [2,18,19] and our earlier studies in this population [20,21].

The final intervention program consisted of two major components. The first component was weekly SMS text message greetings and reminders regarding medication adherence and regular exercise. The second component consisted of short articles on side effect management, medication self-management, stress management, and healthy lifestyle, which were sent via WeChat three times a week. Detailed information on the contents of the articles is shown in Textbox 1. To track patient engagement, the participants would receive 3 multiple choice questions on the information about the articles every other week on WeChat; for example, “what does good medication adherence mean?” with 4 options (>80%, >85%, >90%, and >95% adherence to the prescribed medication).

**Times and titles of articles sent to the participants in the intervention group.**

Week 1

- Common health problems after infection I
- Common health problems after infection II
- Treatment adherence—key to living a healthy life

Week 2

- How to exercise scientifically I
- How to exercise scientifically II
- Take your medicine on time—key to medication adherence

Week 3

- How to have healthy babies for HIV-seropositive men?
- Knowledge about DTG, a new drug
- Something important for men who have sex with men

Week 4

- When to begin HIV treatment, sooner or later?
- To those who are depressed
- Tips on how to quit smoking

Week 5

- Health issues on *Pneumocystis carinii* pneumonia and lactic acidosis
- My life, my choice
- Health issues on bones

Week 6

- Things you need to know about taking medicine I
- Things you need to know about taking medicine II
- Common side effects of medication

Week 7

- I am HIV positive—can I drink alcohol?
- What is drug resistance?
- Consequences of poor medication adherence

Week 8

- Tips for HIV-positive patients on physical checkup I
- Tips for HIV-positive patients on physical checkup II
- His healthy life: a story of an HIV-positive man

Week 9

- Tips about taking medicines
- What to do when you are upset
- Love yourself, love your family

Week 10

- Tips for psychological adjustment
- A brief introduction to opportunistic infection I
- Why you need to take medicine on time every day?

Week 11

- Disclosure of HIV status I
- Disclosure of HIV status II
- Disclosure of HIV status III

Week 12

- Improve your mood, live healthier
- Tips for pregnant women and lactating women
- A brief introduction to opportunistic infection II

Comparatively, the control group received articles on nutrition sent via WeChat three times a week. Each article typically had 1200 Chinese characters and took 3-5 minutes to read through for both intervention and control groups. All the articles were adopted from the authoritative websites of the World Health Organization (WHO) and China’s Centers for Disease Control and Prevention and adapted for PLWH in China.

### Participant Recruitment

The following were the eligibility criteria. The participant should be (1) at least 18 years old, (2) HIV seropositive, (3) on HIV treatment for at least 1 month, and (4) able to read and write. Patients with severe mental illnesses that prohibited them from participating in such intervention were excluded. We recruited participants from the outpatient clinic of the hospital described above. Our research staff approached patients in the waiting areas and invited them to participate in our research project. Those who were interested were taken to a private space for further explanation of the project. Those who met the inclusion criteria and were willing to participate signed an informed consent form before completing a baseline survey. All eligible participants were provided with free breakfast (milk and bread) upon completion of the baseline survey. The study protocol was approved by the Human Subjects Review Board of the School of Public Health, Sun Yat-sen University.

### Randomized Controlled Trial Design

The intervention was delivered as a single-blinded RCT. A total of 62 eligible participants completed the baseline survey and were randomly assigned to the intervention or control group. To ensure that the two groups were balanced in terms of confounding factors, block randomization using SAS statistical software version 9.4 (SAS Institute, Inc., Cary, NC, United States) was conducted [22]. During the 12-week program, participants in the intervention group received a total of 12 SMS text message reminders and 36 WeChat articles. Meanwhile, participants in the control group received a total of 36 WeChat articles; however, they did not receive a SMS text message reminder. Following the HIV/AIDS treatment standard in China, all patients visited their primary health care providers in the designated hospital every 3 months for medication refilling and CD4 testing; thus, we conducted a follow-up survey when the participants returned to the hospital for their medical visit 3 months after the baseline.

### Outcome Measures

All the participants completed the baseline and follow-up surveys using tablets while waiting for their appointments in the outpatient clinic. The survey covered the following domains: participants’ demographic characteristics, medication adherence, mental health, and QoL. Along with patients’ consent, we also obtained data on their CD4 counts from their medical records as a biomarker. Medication adherence was the primary outcome; CD4 count, depression, and QoL were secondary outcomes.

The demographic characteristics of the participants included age, gender, educational level, marital status, sexual orientation, income, and residence (rural or urban). Medication adherence was assessed using the question “In the last 30 days, have you ever missed taking any dose of your HIV medication?” We categorized adherence as a binary variable named “ever missed medication in previous 30 days.” Participants’ depression was measured using the Center for Epidemiological Studies Depression Scale, Chinese version [23,24]. The Cronbach alpha of the scale was 0.9. The total score ranged from 0 to 60 (2-36 in this study); patients with a score of ≥16 were considered to have depressive symptoms. The QoL was measured using the 31-item WHO Quality of Life HIV short form [25]. The total score ranged from 24 to 120 (55-120 in this study); the Cronbach alpha was 0.88.

### Evaluation of the Feasibility and Acceptability of the Study

We conducted semistructured interviews to collect data on the feasibility and acceptability of the intervention and interviewed a total of 31 participants from both the intervention and control groups upon completion of the intervention. The participants were chosen to represent different demographic groups. They were asked about their experience with the intervention, including the design and implementation, and their recommendations on how to improve the program. All interviews were audiotaped and transcribed for content analysis.

### Data Analysis

For the outcome measures of the pilot study, we used the SAS statistical software to perform the analysis. First, we used descriptive statistics to analyze the participants’ characteristics and primary outcomes. Second, we used *t* test (for normally distributed continuous variables), Mann-Whitney U test (for nonnormally distributed continuous variables), and chi-square test (for categorical variables) to compare the demographic characteristics and primary outcomes between the intervention and control groups at baseline and follow-up. Third, we used *t* test to compare the pre-post changes between the intervention and control groups. A two-sided *P* value < 0.05 was considered statistically significant. In total, 85% (53/62) participants finished both the baseline and 3-month follow-ups. For postintervention and pre-post analyses, only 53 participants were included.

For the qualitative data collected from postintervention interviews, we used Nvivo version 10.0 (QSR International Pty Ltd. Doncaster, Victoria, Australia). All audiotaped interviews were transcribed verbatim. Data analysis was started with reading and rereading the transcripts, followed by open-coding the transcripts. Detailed summaries with substantial retention of original quotes were prepared to facilitate further discussion and elaboration among team members. Coding themes and domains were developed by constant comparisons of codes across transcripts and consensus among team members. Coding themes were further analyzed in the original transcripts for consistency and accuracy. Quote excerpts and summaries were then categorized according to participants’ characteristics and coding domains; they were further compared and reviewed for interrelationships and correspondence. A summary report was generated from the qualitative data analysis. This report covered the themes developed in the interview guides as well as new themes identified during the coding process. Each theme was explained using detailed excerpts and summaries of participants’ characteristics.

## Results

### Baseline Characteristics of the Participants

A total of 62 participants completed the baseline survey; of them, 90% (56/62) were male. The mean age of the participants was 28.3 (SD 6.1) years. Among the participants, 76% (47/62) were gay or bisexual, 81% (50/62) had attained high school education or higher, only 8% (5/62) were married, 47% (29/62) were living in urban residences, and only 36% (22/62) had a monthly income >7000 yuan (the average monthly income in Guangzhou being 7425 yuan) [26]. The mean duration since HIV diagnosis was 2.7 years. As shown in Table 1, at baseline, there were no significant differences between the intervention and control groups in terms of demographic characteristics as well as outcome variables of medication adherence, mental health, QoL, and CD4 counts.

### Primary and Secondary Outcomes

Table 2 shows the primary and secondary outcome measures at follow-up and the pre-post changes in these outcomes between the intervention and control groups. There were no significant differences in terms of the primary and secondary outcomes between the intervention and control groups at follow-up. In addition, none of the changes in the primary outcomes were statistically significant between the two groups.

### Feasibility and Acceptability

**Figure 1:**
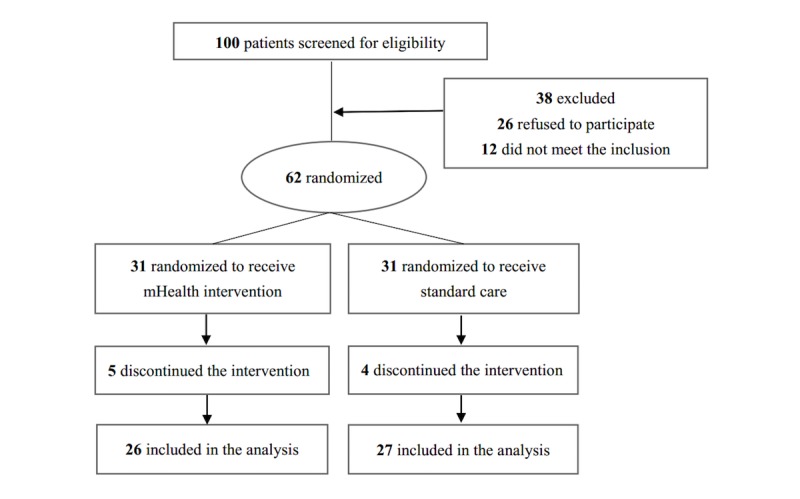
Flowchart of the pilot study.

Figure 1 shows the flowchart of the pilot study. Of all the participants, 85% (53/62) completed the intervention and finished the follow-up survey. Upon completion, 50% (31/62) of the participants were selected for postintervention interviews, with 54% (17/62) being from the intervention group and 46% (14/62) from the control group. They shared the following feedback: (1) The participants were, in general, satisfied with the program and appreciated being cared for. (2) They liked the articles sent to their WeChat account more than the SMS text message reminders. The participants preferred information that was more tailored for the different groups of PLWH, was more personalized, and provided more social support. The biweekly questions to check their reading of the articles were additional burdens for them. (3) The participants were more willing to follow an advice only after having built a trusting relationship with the research staff. They preferred having more interactive communications with the research staff. (4) As most participants had maintained good medication adherence, they were more interested in information to improve their QoL, especially the strategies to reduce anxiety and depression. (5) The participants made specific suggestions regarding the design and content of the WeChat-based program, for example, WeChat-based appointment system and testing result notification and multimedia functions to deliver audio- or video-based interactive programs. Table 3 shows the list of some sample responses from the interviews about feasibility and acceptability.

**Table 1:** Participants’ characteristics and primary outcome measures at baseline.

Characteristics		Total (n=62)	Intervention group (n=31)	Control group (n=31)		*P* value
Age (years), mean (SD)		28.3 (6.1)	29.2 (6.5)	27.4 (5.7)		.26
**Gender, n (%)**					.20	
	Male	56 (90)	26 (84)	30 (97)		
	Female	6 (10)	5 (16)	1 (3)		
**Education, n (%)**					>.99	
	<High school	12 (19)	6 (19)	6 (19)		
	≥High school	50 (81)	25 (81)	25 (81)		
**Sexual orientation, n (%)**					>.99	
	Heterosexual	15 (24)	7 (23)	8 (26)		
	Gay or bisexual	47 (76)	24 (77)	23 (74)		
**Marital status, n (%)**					.35	
	Married	5 (8)	4 (13)	1 (3)		
	Unmarried	57 (92)	27 (87)	30 (97)		
**Residence, n (%)**					.20	
	Urban residence	29 (47)	17 (55)	12 (39)		
	Rural residence	33 (53)	14 (45)	19 (61)		
**Monthly income (yuan), n (%)**					.30	
	＜3000	15 (24)	5 (16)	10 (32)		
	3000–7000	25 (40)	14 (45)	11 (35)		
	＞7000	22 (36)	12 (39)	10 (32)		
Duration since HIV diagnosis (years), mean (SD)		2.7 (2.4)	3.1 (2.2)	2.3 (2.5)		.24
CD4^a^ cell counts (cells/μL), median (interquartile range)		392 (277-517)	380 (283-542)	414 (260-513)		.89
Missed medication within the last 30 days, n (%)		5 (8)	3 (10)	2 (6)		>.99
Depression, mean (SD)		16.9 (9.4)	15.8 (9.4)	18.0 (9.3)		.36
Quality of Life (total scores), mean (SD)		83.4 (12.7)	84.3 (14.2)	82.6 (11.2)		.60

^a^CD4: cluster of differentiation 4.

**Table 2:** Postintervention endpoint analyses and pre-post analyses of the primary outcomes.

Characteristics	Total (n=53)	Intervention group (n=26)	Control group (n=27)	*P* value
CD4^a^ cell counts (cells/μL), median (interquartile range)	399 (270-564)	379 (254-570)	401 (272-524)	.89
CD4 change (cells/μL), mean (SD)	5 (111)	11 (122)	0 (101)	.71
Missed medication within the last 30 days, n (%)	3 (6)	2 (8)	1 (4)	.39
Depression, mean (SD)	16.7 (10.1)	15.5 (9.1)	17.9 (11.1)	.38
Changes in depression change, mean (SD)	−0.28 (8.15)	−0.42 (7.04)	−0.15 (9.22)	.90
QoL^b^, mean (SD)	82.2 (13.7)	85.0 (13.2)	79.5 (13.8)	.15
Changes in QoL, mean (SD)	−2.1 (9.9)	−0.7 (10.6)	−3.5 (9.3)	.32

^a^CD4: cluster of differentiation 4.

^b^QoL: Quality of Life.

**Table 3:** Summary of the qualitative analysis on the feasibility and acceptability evaluation.

Domains	Feasibility and acceptability assessment questions	Typical answers
WeChat versus short message service (SMS) text messages	How do you like the articles sent via WeChat and reminders via SMS text messages?	“I think they are helpful, I learned a lot about the disease that I had not known before.” “All of my friends are using WeChat instead of short message now. So I prefer the articles sent via WeChat.”
Content of the articles	What topics are you most interested in?	“I follow the latest progress of HIV treatment.” “I have been feeling awful since I realized I was infected. I feel my life is ruined. Anything to help me feel better would be helpful.” “In my opinion, the articles are not very targeted. If you can tell us something specific for us gay patients, it will be useful.”
Format or style of the articles	What kind of format or style of the articles do you like?	“There is too much information on the internet. Sometimes it can be very confusing. I hope the articles can be professional and authoritative.” “Well, reading words is kind of boring, it’d be better if I can listen to it, and video would be perfect.”
Intervention adherence	To what extent did you read the articles and messages?	“I use WeChat every day; reading articles on WeChat is convenient for me, so I read most of them. But I often missed messages via SMS as I do not use SMS much. Questions you asked are difficult for me. If I do not know the answer immediately, I do not answer them.” “I have subscribed many Subscription Accounts; they send me articles every day, so I do sometimes miss some of your articles.”
Satisfaction	Are you satisfied with our intervention?	“Of course, I am so pleased that someone like you cares about people like me.”
Suggestions	Do you have any suggestions for our mHealth intervention?	“If I can make an appointment and get my testing results via the system, it will save me a lot of time.” “I didn’t have any motivation to read your articles. Maybe you can have more appealing titles for your articles.” “Repeated messages became burdens. I do not trust machine. What I really need are communication and interaction with real people.”

## Discussion

This pilot study represents one of the first efforts to develop an mHealth intervention for improving medication adherence in and QoL of PLWH in China. The outcome analyses indicated nonsignificant results in this study. Studies on prior SMS text message-based interventions to improve medication adherence in PLWH in middle- and low-income countries have mostly reported nonsignificant results, with few studies reporting significant viral suppression [8,27]. The following reasons might explain the nonsignificant results observed in this pilot study. First, a ceiling effect existed in the primary outcome of ART adherence; 92% of the participants had good adherence at baseline. Such high adherence rate might be due to the fact that 64.5% (40/62) of the participants were men who have sex with men (MSM) aged under 30 years, with a mean age of 28.3 years, and 38.7% (24/62) participants had attained college education or higher. The characteristics of our participants, being young and highly educated, were similar to those of the participants in earlier studies on MSM in China, a group with high rates of medication adherence [28]. Second, in the RCT, the control group received HIV-related nutrition information at the same frequency as the intervention group. Their feedback after the project has suggested that they were very interested in such information, and they became more cautious of their HIV self-management including medication adherence. Third, we observed a limited interaction between health care providers and patients as well as among patients during the intervention. Thus, the intervention mostly took effect at an individual level, with minimal effects at health care and community levels. Fourth, our SMS text message+WeChat systems could not track whether the participants had actually opened or read the information sent by us. Thus, we could not measure patient engagement or intervention exposure. From the postintervention feedback, we learned that some participants did not read all the articles sent by us, suggesting the need for better content design and innovative strategies to track and engage participants. Last, the small sample size of the pilot study might limit the power to detect significant differences hypothesized by us.

Despite these nonsignificant effects, this study showed feasibility and acceptability of the mHealth intervention in PLWH in China. Patients generally welcomed articles sent via WeChat and made specific recommendations to improve the intervention design and implementation. For example, they preferred receiving information via WeChat instead of SMS text messages; they welcomed more appealing design with multimedia functions. Furthermore, they expressed a strong need for programs that help combat depression and anxiety.

Based on the data collected from the pilot study, we proposed the following modifications for mHealth interventions for PLWH. First, the programs need to address the primary concern of the participants. For example, for PLWH in urban areas in China, poor mental health is the primary challenge that they face on a daily basis. It should be a priority in future health services provided to PLWH. Second, mHealth programs must go beyond traditional usability design and be more user centered. Because most mobile phone users are overloaded with information, the user experience is critical. For example, more tailored design with personalized feedback must be utilized. Third, theory-guided and evidence-based mHealth interventions should also incorporate tracking systems to measure user engagement and intervention exposure. Fourth, when testing the intervention, we need to recruit a diverse sample of PLWH of different age groups, educational levels, and transmission modes. Fifth, building a trust-based relationship between the participants and research staff of the program through personalized interactive communication is important for intervention adherence and participant retention. Finally, the efficacy trial must have a sufficient sample size and multiple follow-ups for the observation of intervention effects [29].

With the high penetration rates of mobile phones, mHealth interventions for PLWH have become more popular [3,26,30]. To date, the evidence on the effectiveness of mHealth interventions to promote medication adherence in PLWH has been preliminary [14,18,19,31,32] and the clinical evidence about viral suppression has been minimal [7,8,27]. The experiences from this study provided valuable inputs on the design and implementation of mHealth interventions for PLWH in middle- and low-income countries. Our team has revised the intervention protocol based on the experience from this pilot study, and a larger RCT is underway. We call for more evidence-based mHealth interventions with rigorous designs to serve the vulnerable population of PLWH in middle- and low-income countries.

## Appendix

Multimedia Appendix 1 CONSORT‐EHEALTH checklist (V 1.6.1).

## Abbreviations

ART: antiretroviral therapy
CD4: cluster of differentiation 4
MSM: men who have sex with men
PLWH: people living with HIV
QoL: quality of life
RCT: randomized controlled trial
SMS: short message service
WHO: World Health Organization
